# Bioactive Compounds Produced by Strains of *Penicillium* and *Talaromyces* of Marine Origin

**DOI:** 10.3390/md14020037

**Published:** 2016-02-18

**Authors:** Rosario Nicoletti, Antonio Trincone

**Affiliations:** 1 Council for Agricultural Research and Agricultural Economy Analysis, Rome 00184, Italy; 2 Institute of Biomolecular Chemistry, National Research Council, Pozzuoli 80078, Italy; antonio.trincone@icb.cnr.it

**Keywords:** bioactive metabolites, chemodiversity, marine fungi, *Penicillium*, *Talaromyces*

## Abstract

In recent years, the search for novel natural compounds with bioactive properties has received a remarkable boost in view of their possible pharmaceutical exploitation. In this respect the sea is entitled to hold a prominent place, considering the potential of the manifold animals and plants interacting in this ecological context, which becomes even greater when their associated microbes are considered for bioprospecting. This is the case particularly of fungi, which have only recently started to be considered for their fundamental contribution to the biosynthetic potential of other more valued marine organisms. Also in this regard, strains of species which were previously considered typical terrestrial fungi, such as *Penicillium* and *Talaromyces*, disclose foreground relevance. This paper offers an overview of data published over the past 25 years concerning the production and biological activities of secondary metabolites of marine strains belonging to these genera, and their relevance as prospective drugs.

## 1. Introduction

For a long time fungi have been considered as a fundamentally terrestrial form of life. In the past few decades, this concept has started to be revised based on the emerging evidence that these microorganisms are also widespread in the marine habitat. New species recovered from marine substrates are reported repeatedly, which makes a reliable estimate of their actual number quite problematic [1]. Attention of researchers in the field often tends to be focused on the obligate marine species, defined for their ability to grow and sporulate exclusively in a marine habitat [2]. However, it is a matter of fact that many species found at sea are already known from terrestrial contexts, which makes their placement in a “facultative” category more appropriate. It is obvious that the mere isolation from a marine substrate does not imply a real adaptation of a fungus to develop in those particular conditions. Nevertheless, this aspect becomes secondary when considering discovery and exploitation of bioactive compounds, and in view of this objective the ecological versatility of the facultative marine fungi introduces them as being among the most valuable natural resources, deserving to be better characterized through more detailed genetic and biochemical analyses [3].

The issue of bioactive compound production is fundamental in understanding the complex ecological relationships established among and between sea-inhabiting organisms and microorganisms, and presents human nutritional implications due to the possibility that such fungal strains contaminate sea food, and their metabolites eventually act as mycotoxins [4,5]. However, the pharmaceutical industry can be regarded as the application field where products from marine fungi have the most substantial impact, since many of them have entered the clinical pipeline in view of being exploited as novel drugs. Quantitative considerations about fruitfulness in the discovery of new metabolites show that the number of compounds obtained from marine-derived fungi is increasing at a high rate. From a total of about 270 known before 2002, investigations in the field have added more than 800 such products up to 2010 [6], as a result of the availability of bioassay-guided fractionation systems, the accessibility to higher field NMR and mass spectrometers, and the development of the so-called hyphenated spectroscopy technologies (HPLC-MS, HPLC-NMR, *etc.*) [7]. The recent combining of natural product chemistry and metabolomic approaches in drug discovery can certainly contribute to the development of new leads from marine derived fungi [8].

Within the facultative marine fungi, species of *Penicillium* and *Talaromyces* are particularly known for their ability to produce important bioactive compounds. This paper offers an overview of the literature issued in the past 25 years concerning production and biological activities of secondary metabolites of marine strains belonging to the above genera, and their relevance as prospective drugs. Our review basically considers strains obtained from marine sources in a topographic sense, thus possibly including strains/species whose occurrence at sea is merely incidental. Conversely, we did not treat isolates from mangrove plants and their rhizosphere, whose connection with the sea is more remote, and probably deserve a dedicated review. As for the compounds, this overview does not consider primary metabolites mentioned in the cited references, including ergosterol and structurally related compounds [9,10]. Additional exclusions concern other common compounds which often represent intermediates in the synthesis of more complex secondary metabolites, such as orsellinic acid [11,12], and products obtained from mutant strains [13,14,15], or through co-cultivation of two or more strains [16].

## 2. *Penicillium* and *Talaromyces*: An Extraordinary Source of Bioactive Compounds

The Ascomycetous genus *Talaromyces* (Eurotiomycetes, Trichocomaceae) was initially designated to comprise the teleomorphs of a number of biverticillate *Penicillium* species. However, following the principle “one fungus—one name” recently affirmed in fungal taxonomy, by which a single holomorphic denomination is to be adopted for species presenting two alternating stages in their life cycle [17], the concept of *Talaromyces* has been recently extended to include all species in the *Penicillium* subgenus *Biverticillium* [18], while the name *Penicillium* is conserved *sensu stricto* for species belonging to the subgenera *Aspergilloides*, *Furcatum*, and *Penicillium*, for their associated *Eupenicillium* teleomorphs, and for species previously classified in a few related genera [19]. Information concerning production of secondary metabolites also supports the separation of the two genera in distinct monophyletic groups based on DNA sequencing [20], and the accumulation of novel data provides a remarkable contribution under the taxonomic viewpoint, particularly in view of a correct species ascription of the many strains which are provisionally reported as *Penicillium*/*Talaromyces* sp. [18,21].

Traditionally, species in the genus *Penicillium* and *Talaromyces*, which are fundamentally saprophytic and ubiquitary, have been regarded as a fruitful investigational ground for the finding of novel bioactive compounds, leading to the discovery of blockbuster drugs, such as penicillin [22] and the anticholesterolemic agent compactin [23], miscellaneous antitumor products [24], and mycotoxins contaminating food [25]. Most of these fundamental studies were carried out on strains from soil and food commodities. Thus, in a way it is not surprising that the number of bioactive compounds is continuously increasing when a so far inadequately explored context such as the sea has become the subject of systematic investigations. This inference is particularly valid for the species treated in this paper, considering that in a recent review *Penicillium* is reported as the second most common genus of marine fungi [26]. In our overview we recorded over 550 compounds, or compound families, from a total of about 150 strains belonging to 39 species of *Penicillium* and five species of *Talaromyces* (Table 1). Unclassified strains, referred to as *Penicillium* sp. or *Talaromyces* sp., represent a remarkable share (*ca.* 38%), which implies that the number of marine species within these genera is destined to increase when and if more work is carried out on some of these strains, eventually leading to their correct species ascription. With reference to this taxonomic aspect, the characterization of two novel species, *Penicillium marinum* [25] and *Penicillium dravuni* [27], deserves to be particularly mentioned.

Concerning sources, 49 strains were recovered from inanimate substrates, mainly sediment and water samples. As for living organisms, sponges appear to be the most widely reported hosts with 33 strains, confirming recent evidence of their significant interaction with fungi [176,177], while the other sources are represented by a disparate set of animals and plants including shellfish, gorgonians and corals, a few tunicate, urchin and fish species, brown, red and green algae, and a single Angiosperm plant (*Zostera marina*).

**Figure 1:**
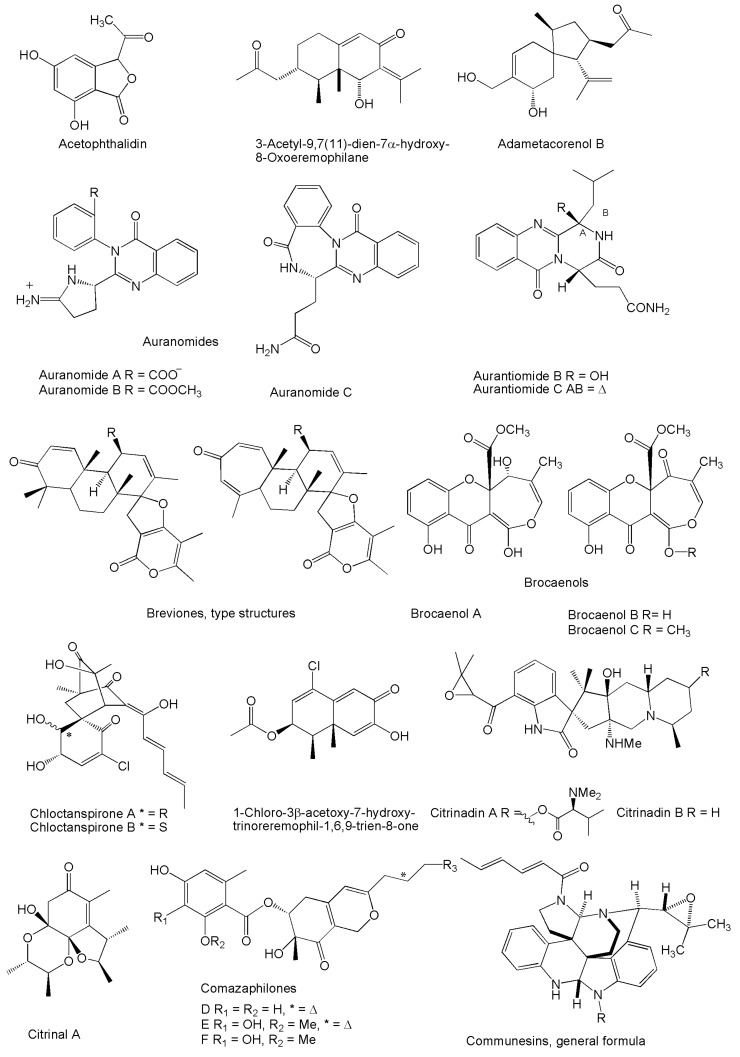

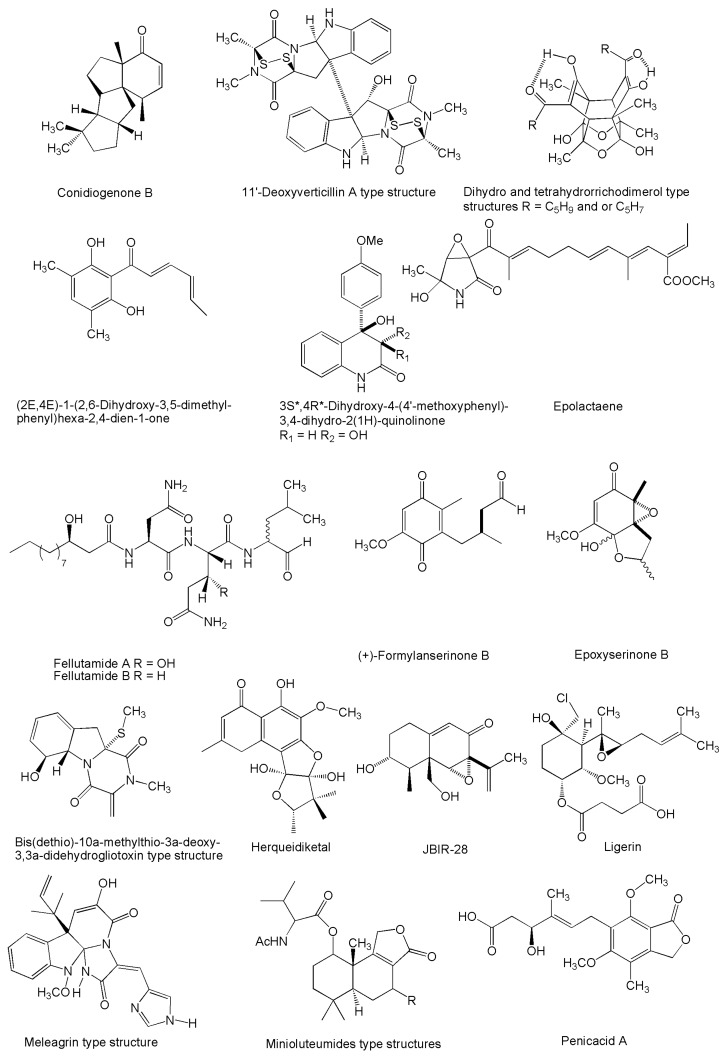

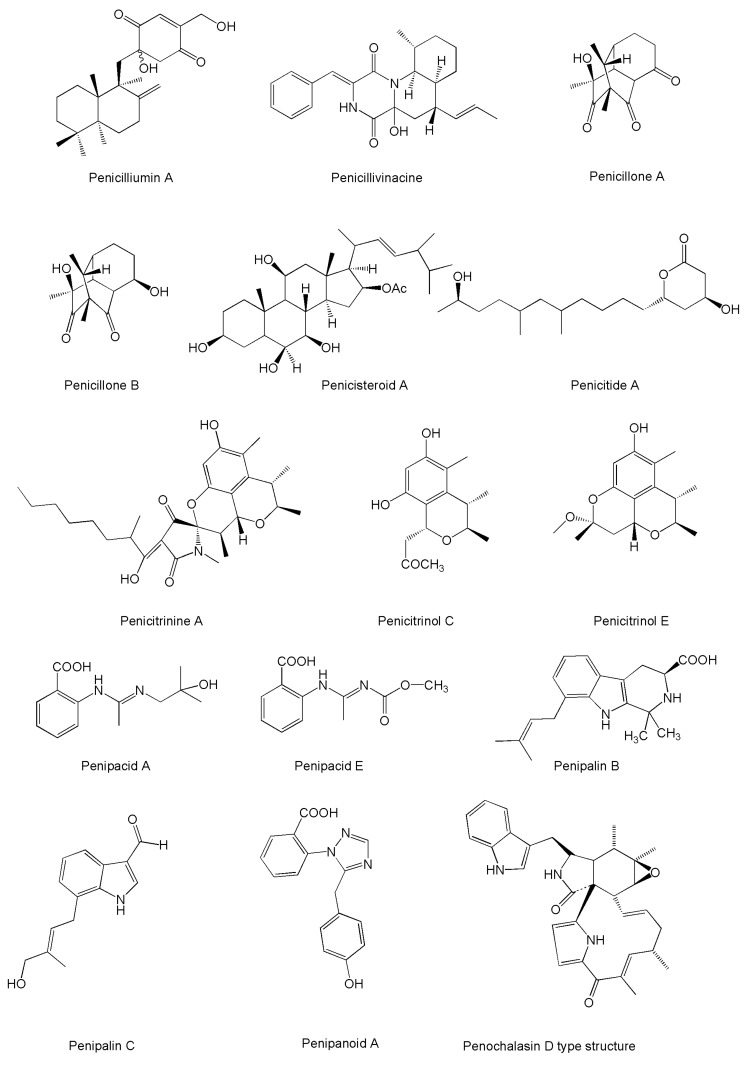

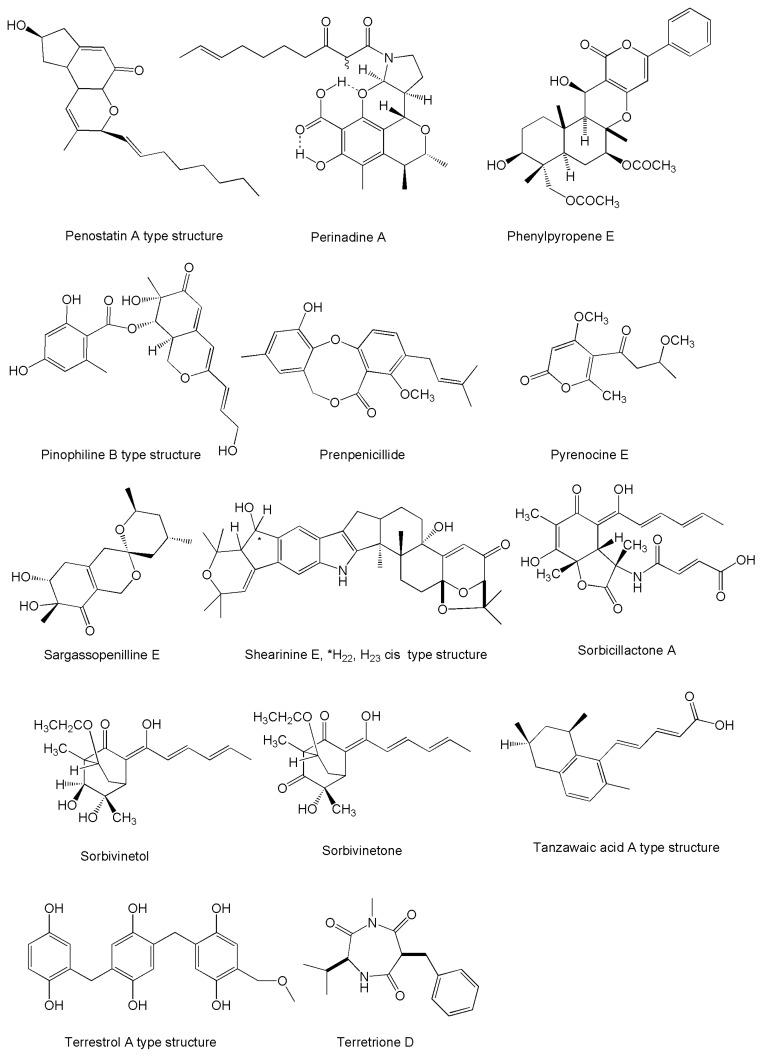
Structures of novel compounds produced by marine *Penicillium*/*Talaromyces* strains displaying inhibitory properties against mammalian tumor cell lines. For the sake of space, compounds produced in series of two or more analogs are presented as a single or type structure.

**Figure 2:**
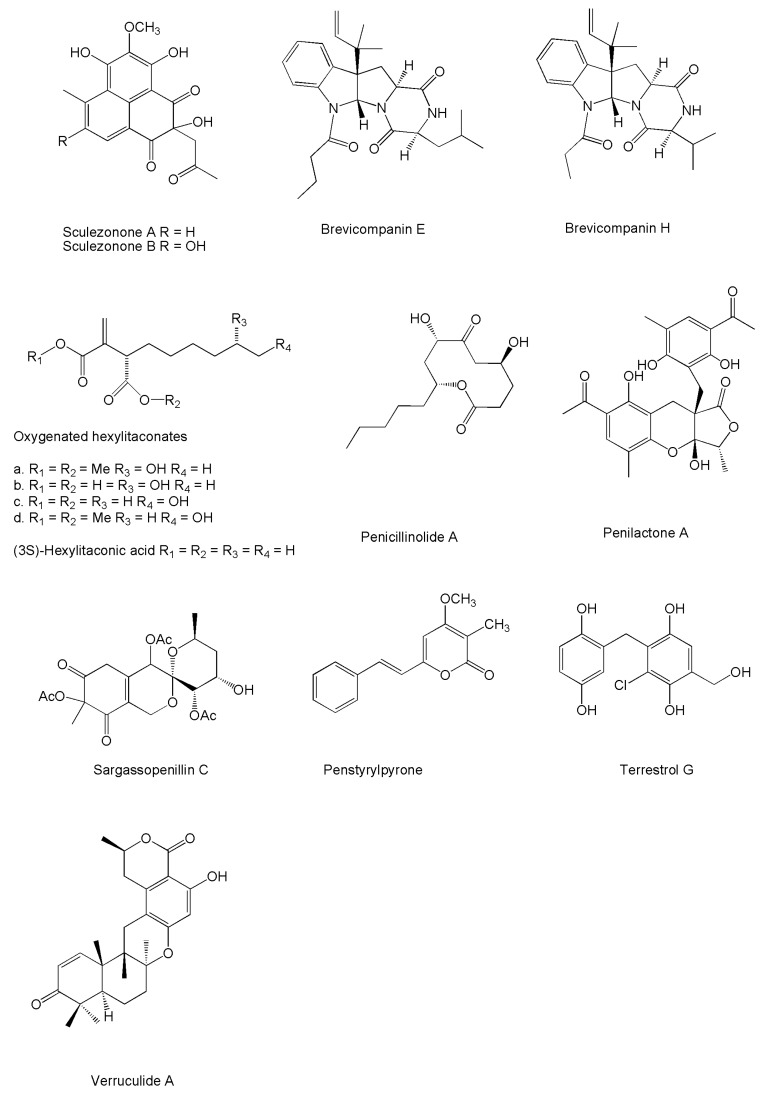
Structures of novel compounds produced by marine *Penicillium*/*Talaromyces* strains displaying anti-inflammatory effects.

**Figure 3:**
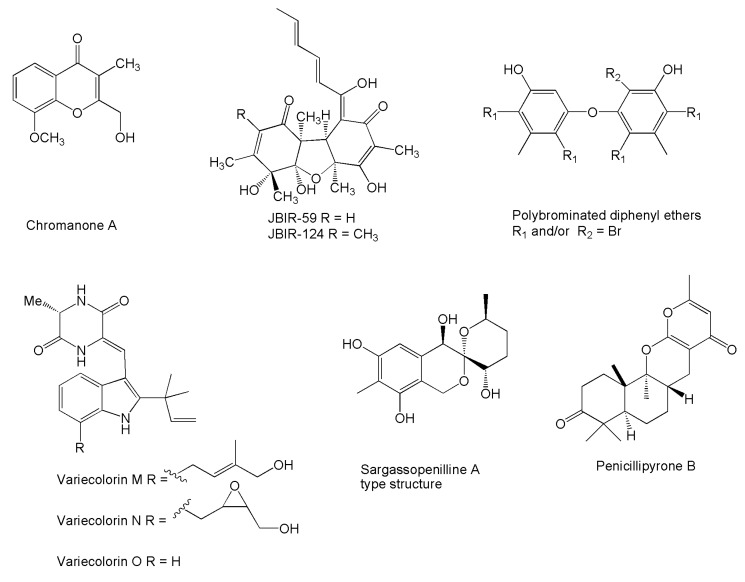
Structures of novel compounds produced by marine *Penicillium*/*Talaromyces* strains reported for ROS-scavenging properties.

**Figure 4:**
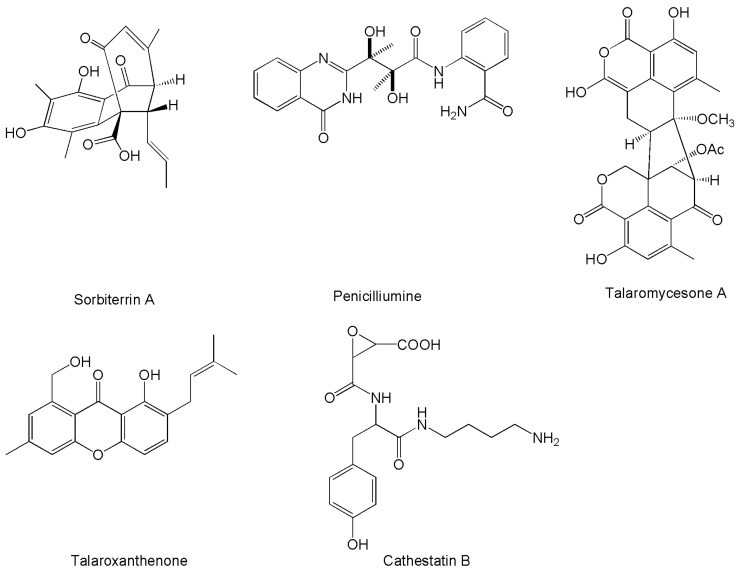
Structures of novel compounds produced by marine *Penicillium*/*Talaromyces* strains with enzyme-modulatory activities.

**Figure 5:**
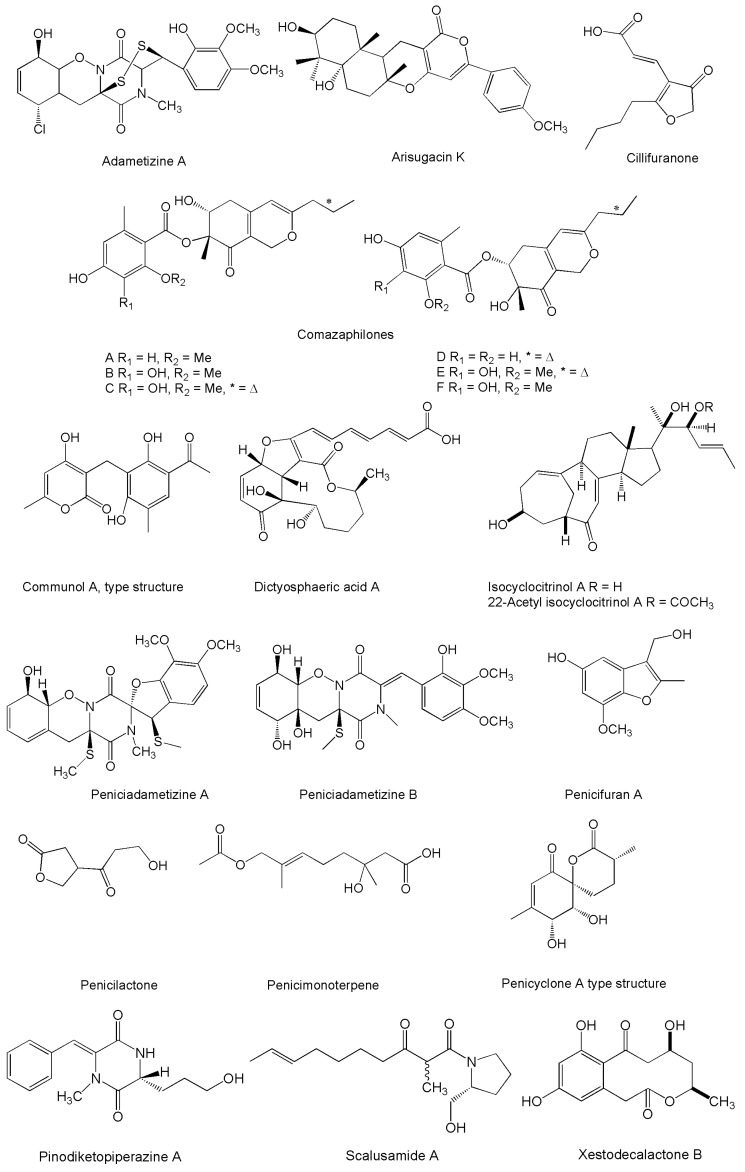
Structures of novel antibiotic compounds produced by marine *Penicillium*/*Talaromyces* strains.

**Figure 6:**
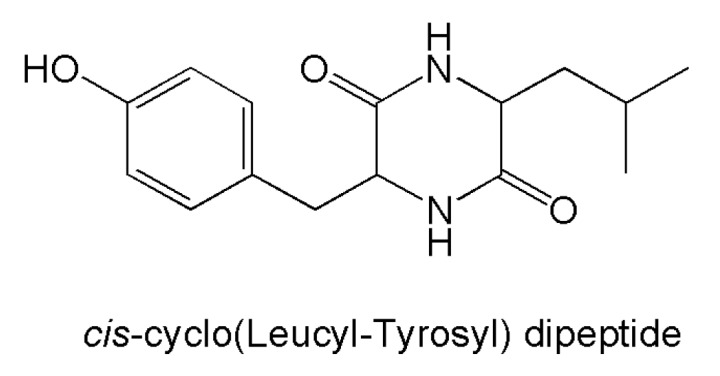
Structure of *cis*-cyclo(leucyl-tyrosyl) dipeptide.

**Figure 7:**
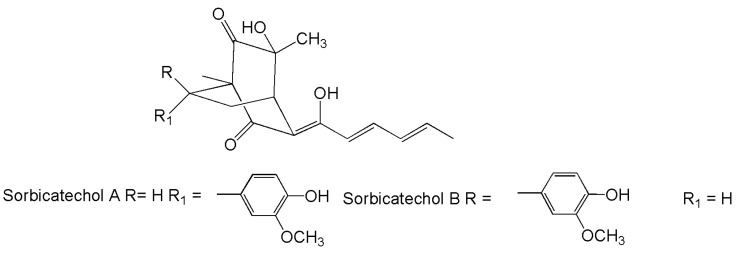
Structures of sorbicathecols.

**Figure 8:**
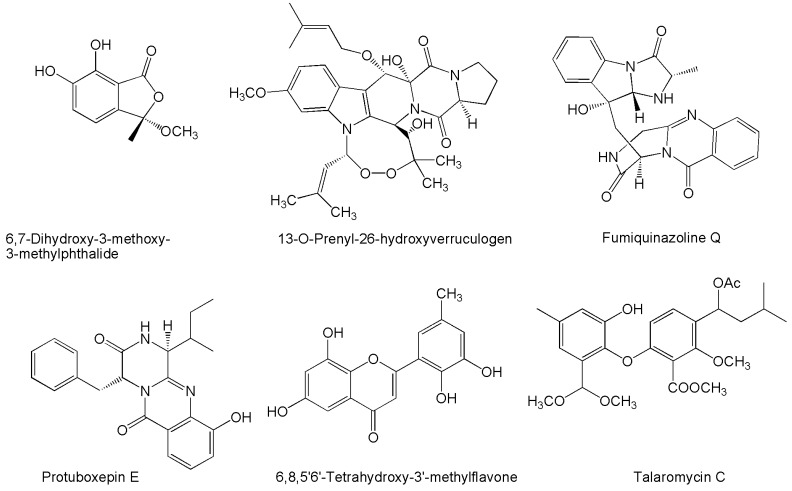
Structures of novel compounds produced by marine *Penicillium*/*Talaromyces* characterized for miscellaneous bioactive effects.

About half of the compounds listed in Table 1 (underlined names) were first characterized in strains from marine sources. This remark not only indicates, once again, that sea is a fruitful context for drug discovery, but also introduces a point of view that the ecological relationships established with marine organisms by species which are ordinarily reported from terrestrial environments may somehow address the biochemical pathways toward the synthesis of some peculiar compounds. In this sense, it must be emphasized that a number of unusual molecular structures have been first elucidated from this biological material (Figure 1, Figure 2, Figure 3, Figure 4, Figure 5, Figure 6, Figure 7 and Figure 8).

The rest of the compounds itemized in Table 1 were first extracted and characterized for their bioactivity from terrestrial fungal strains, and a few of them are already known as drugs, or drug prospects. Particularly, mycophenolic acid is famous as the first known fungal antibiotic, discovered as a product of a strain of *Penicillium brevicompactum* even before the start of the 20th century [178], although its real structure was elucidated only after a few decades [179]. This compound displayed notable antibiotic, antiviral, and cytostatic properties, and has found consistent medical application as an immunosuppressive drug in the derivate form of mycophenolate mofetil [180,181]. First extracted from a strain of *Penicillium griseofulvum* [182], but later detected in many congeneric species, griseofulvin gained notoriety as an antimycotic drug, and more recently is being considered for its antitumor properties [183]. Again with reference to their antibiotic/cytotoxic properties, gliotoxin and the chaetoglobosins were first characterized from unrelated fungi, respectively *Gliocladium fibriatum* [184], and *Chaetomium globosum* [185]. However, both these compounds were later detected in a few *Penicillium* species, and considered for a series of interesting effects on human tumor cells [24]. Finally, 3-*O*-methylfunicone was first identified in connection with the antagonistic/mycoparasitic aptitude of the producing strains of *Penicillium pinophilum* (=*Talaromyces pinophilus*) [186,187], and later thoroughly characterized for its cytostatic properties on a number of human tumor cell lines, based on effects on cytoskeletal organization, cell cycle progression, the expression of pro-apoptotic genes, the inhibition of markers of tumor progression, and other mechanisms suppressing cell proliferation/migration [188,189,190,191,192,193,194,195,196]. Moreover, remarkable activity as a DNA polymerase inhibitor makes it one of the few known natural compounds displaying this particular effect [83]. Taken together, these valuable biological properties introduce 3-*O*-methylfunicone as a candidate molecule for more accurate clinical investigations in view of its development as an antitumor drug [197].

With a complex structure based on highly oxygenated, bicyclic and tricyclic frameworks, sorbicillinoids are a class of compounds which include over 50 members [198]. Their name derives from sorbicillin, which was first extracted from a terrestrial strain of *Penicillium notatum* (=*Penicillium chrysogenum*) [199]. However, a significant number of analog compounds showing peculiar structural models and consistent bioactive properties have been reported from marine fungi. Producing strains cited in this review were ascribed to a few unrelated species, such as *Penicillium citrinum*, *Penicillium crustosum* and *Penicillium commune* (Table 1). However, authorities in *Penicillium* taxonomy consider these strains to have been probably misidentified, by reason of strict evidence that these products are typical of *P. chrysogenum* and allied species [200].

Other products listed in Table 1 are best known for their noxious effects as mycotoxins contaminating foodstuffs. This is the case of cyclopiazonic acid, verrucosidin, fumitremorgin, and a few related tremorgenic toxins, secalonic acids, and particularly of citrinin and patulin [25]. Actually, the concern for dietary intake of mycotoxins produced by *Penicillium* strains has recently reached seafood, and specific investigations are being carried out in order to better assess the associated risk for consumers [54,128,201].

## 3. Bioactivities of Novel Compounds

By reason of the quite short time having elapsed after their discovery, most of the novel compounds obtained from marine strains of *Penicillium/Talaromyces* have been characterized for their biological properties and mechanisms of action only at a preliminary stage. In this regard, the largest category of bioactivity is undoubtedly represented by the cytotoxic/antiproliferative products (Table 2, Figure 1). In fact, assays on human or mammalian cell lines have become widespread following the recent trend to identify new natural antitumor compounds [202], and in view of pursuing this general aim there is a tendency to inappropriately consider these terms as synonyms [203]. Even if such a frequent semantic impropriety does not affect the significance of preliminary bioactivity screenings, the possible relevance of these molecules as antitumor prospects can be introduced only when a further characterization of their cytological effects is accomplished, which quite notably reduces the number of compounds deserving to be further examined in this review.

Besides consistent pro-apoptotic effects on human promyelocytic leukemia cells, the indole diterpenoid alkaloid shearinine E has been characterized for its ability to inhibit the malignant transformation of mouse epidermal cells (JB6P + Cl41) experimentally induced by the epidermal growth factor in the anchorage-independent transformation assay [74].

Another alkaloid with a unique spiro skeleton, penicitrinine A, was found to induce some typical modifications in melanoma cells undergoing apoptosis, such as shrinkage, fragmentation, and chromatin condensation. Assays based on annexin-V/PI double staining showed that apoptosis occurred at a higher rate than control cells treated with the chemotherapeutic drug 5-fluorouracile. Apoptosis followed the mitochondrial pathway, as indicated by down-regulation of the anti-apoptotic gene Bcl-2 and concomitant up-regulation of the pro-apoptotic gene Bax, and the ratio of Bcl-2/Bax expression, which decreased with increasing concentrations of the compound. Anti-metastatic dose-dependent effects were also observed as a result of suppression of invasiveness and inhibition of cell migration, which is an ill-fated tendency of melanoma cells. These latter effects are related to a down-regulation of matrix metalloproteinase (MMP-9) expression along with up-regulation of its inhibitor glycoprotein TIMP-1 [111].

Besides selectively suppressing cell growth and proliferation in five human cancer cell lines, pinophilins displayed a strong inhibitory activity on DNA polymerases of the A-, B-, and Y-families, particularly against DNA pol-*α* and -κ. The inhibitory effect was selective, since it was not observed on normal human cells (dermal fibroblasts and umbilical vein endothelial cells), possibly because their DNA replication rates are significantly slower than those of cancer cells [122]. Bioactivity as DNA polymerase inhibitors had been previously showed for two phenalenone compounds, the sculezonones A–B. Particularly, both compounds inhibited bovine DNA pol-α and -γ, and moderately affected the activity of DNA pol-ε. Moreover, DNA pol-β was inhibited by sculezonone A, and just weakly influenced by sculezonone B [205]. Another DNA polymerase inhibitor, the γ-lactam compound epolactaene [36], not only was effective on bovine DNA pol-α and rat DNA pol-β, but also disclosed inhibitory properties against human DNA topoisomerase II [206], which is a very important biomolecular mechanism considered in prospecting for antitumor drugs [207]. Consistent neuritogenic effects [36] make this compound even more valuable in view of possible medical applications.

Some degree of neural stimulation was also pointed out for the fellutamides. In fact these tripeptides were characterized as potent enhancers of the release of the nerve growth factor (NGF) from fibroblasts and glial-derived cells [208]. This effect results from the inhibition of proteasome catalytic activity, which leads to increased NGF gene transcription [209]. For different respects, neuroprotective properties have been reported for sorbicillactone A, which impaired the negative effects of important neurotransmitters such as l-glutamic acid and serotonin [62], and brevicompanines E and H (Figure 2), which have been characterized as neuroinflammation modulators [87]. More in detail, in BV2 mouse microglial cells brevicompanine E was found to inhibit production of tumor necrosis factor-α (TNF-α), interleukin-1β (IL-1β), inducible nitric oxides (iNOS), and cyclooxygenase-2 (COX-2), and to reduce the DNA binding activity of the oncogenic nuclear factors AP-1 and NF-κB. Nuclear translocation of the latter was also inhibited, together with IκBα degradation, and Akt and c-Jun NH_2_-terminal kinase phosphorylation [210]. Similar anti-inflammatory effects were also evidenced in murine peritoneal macrophages for novel ester derivatives of hexylitaconic acid [115], and for penicillinolide A [138] (Figure 2). Weak NF-κB inhibitory properties were again reported from penilactone A [125], while sargassopenilline C has been found to inhibit the transcriptional activity of AP-1 [160] (Figure 2). Finally, and again in BV2 cells, *2E*,*4Z*-tanzawaic acid D was found to inhibit the production of iNOS [158].

Penstyrylpyrone (Figure 2) is another product reported for considerable anti-inflammatory activity deriving from inhibition of the expression of iNOS and COX-2, reduction of TNF-α and IL-1β production, suppression of phosphorylation and degradation of IκB-α, and of NF-κB nuclear translocation and DNA binding activity. These effects were found to be associated with the expression of heme oxygenase 1 (HO-1), an enzyme releasing anti-inflammatory degradation products during heme catabolism. Ultimately, these anti-inflammatory effects lead to a competitive inhibition of the protein tyrosine phosphatase 1B (PTP1B), which is known to play a major role in the negative regulation of insulin signalling. Therefore, this compound was introduced as a prospect therapeutic drug for the treatment of type II diabetes [140]. Inhibitory properties towards PTP1B were also disclosed for penostatins A–C [211], while verruculides A and B respectively displayed a strong and a moderate effect against this enzyme [173]. Finally, a moderate effect as an inhibitor of tyrosine kinases was reported for terrestrol G (Figure 2) [64].

Another target in the search for antitumor products is represented by the reactive oxygen and nitrogen species (ROS), whose excessive production results in oxidative stresses, DNA damage, and inflammation, as well as contributing to tumor initiation and promotion. Consequently, scavenging of the physiologically relevant ROS represents an effective strategy in preventing tumor initiation and promotion. Chromanone A (Figure 3) was characterized as a strong OH scavenger, which also dramatically inhibits the degree of DNA fragmentation. Moreover, it was able to act, in a dose-dependent manner, as a potent inhibitor of cytochrome P4501A, and as an inducer of GSH (cytosolic thiol) and GST enzymes, which both help in the destruction of peroxides, free radicals, and other xenobiotics [86].

Radical scavenging effects were also reported for compound JBIR-59 (Figure 3), on account of its protective effects against l-glutamate toxicity in neuronal hybridoma N18-RE-105 cells [99]. A few more products (Figure 3) have been characterized as free radical scavengers based on their activity against 1,1-diphenyl-2-picrylhydrazyl (DPPH), such as the terrestrols [64], 4,6,4′,6′-tetrabromo-3,3′-dihydroxy-5,5′-dimethyldiphenyl ether and 4,6,2′,4′,6′-pentabromo-3,3′-dihydroxy-5,5′-dimethyldiphenyl ether [105], the variecolorins [102], compound JBIR-124 [100], and sargassopenillines A and E [160]. Further indirect antitumor effects resulting from detoxification of xenobiotics have been proposed for the meroterpenoid penicillipyrone B (Figure 3) for its ability to induce the enzyme quinone-reductase, which is involved in the reduction of electrophilic quinones [151].

Other enzyme modulatory activities are relevant in human medicine for the treatment of a number of complex diseases. This is the case for Alzheimer’s disease, where compounds performing acetylcholinesterase inhibition can be considered in view of possible therapeutic use [212]. In this regard, moderate activity has been reported for products such as sorbiterrin A [65], and penicilliumine [149], while more potent effects have been evidenced for talaromycesone A and talaroxanthenone [161] (Figure 4). The latter compound also showed activity as an inhibitor of phosphodiesterase, which is a target in the treatment of inflammatory processes involved in pulmonary diseases [161]. Besides general activity as inhibitors of proteases, such as papain, ficin, and bromelain, the cathestatins, particularly cathestatin B (Figure 4), were introduced for possible useful effects in the treatment of osteoporosis deriving from the inhibition of bone collagen degradation, and the suppression of calcium release [35]. Finally, along the lines of their more famous analog mycophenolic acid, the penicacids (Figure 4) were investigated for their immunosuppressive properties, and found to possess appreciable inhibitory effects towards inosine-monophosphate dehydrogenase [124].

Antibiotic properties have been assessed for a number of novel compounds (Figure 5) against the bacterial and fungal species indicated in Table 3. Besides these general inhibitory effects, some peculiar mechanisms of action were evidenced for the dipeptide *cis*-cyclo(leucyl-tyrosyl) (Figure 6), which inhibits biofilm formation by *Staphylococcus epidermidis* without interfering with bacterial growth [144], and herqueidiketal, which was characterized for inhibitory properties against sortase A of *Staphylococcus aureus*. Since sortases are absent in mammals, this biochemical effect may be relevant for the development of novel antibiotics [130]. Moreover, the above-mentioned proteasome inhibitory effects by fellutamide B were again observed on *Mycobacterium tubercolosis*, introducing this peptide as a prospect drug to be more thoroughly investigated against such a deadly pathogen [213].

A few compounds displayed consistent effects against important viruses, such as the influenza virus A (H1N1) and HIV-1. Particularly, the cytopathic effects induced by the former in MDCK cells were found to be inhibited by sorbicathecols A–B (Figure 7) [153], while the latter was impaired by brevione F, which inhibited its replication in C8166 cells [92], and sorbicillactone A, which inhibited the expression of viral proteins and protected H9 cells (human T lymphocytes) against cytopathic effects [62].

Finally, some miscellaneous bioactive effects can be mentioned for a few compounds (Figure 8).

In the search for novel products to be used as additives in antifouling paints used as protective coats for ships’ hulls, potent activities against the larval settlement of barnacles (*Balanus amphitrite*) were evidenced for 6,8,5′6′-tetrahydroxy-3′-methylflavone [136], and talaromycin C [162].

Widely considered as a model organism used to test the toxicity of chemicals, brine shrimp (*Artemia salina*) has been employed for demonstrating the toxic effects of products such as 6,7-dihydroxy-3-methoxy-3-methylphthalide [132], 13-*O*-prenyl-26-hydroxyverruculogen [148], adametizine A [155], the peniciadametizines [156], and the communesins [51]. The latter represent a numerically expanding series of cytochalasan alkaloids which have been also introduced to some extent for cytotoxic/antiproliferative properties (Table 2), and insecticidal effects resulting after oral administration to silkworms [215]. Insect neurotoxicity was also observed in assays carried out on larvae of the bluebottle fly (*Calliphora vomitoria*) [94]. Moreover, in a study employing a zebra-fish model, communesin I and two more novel compounds, fumiquinazoline Q and protuboxepin E, were reported for cardiotonic effects, as well as some extent of vasculogenetic properties assessed with reference to both number and length of vessels [168].

## 4. Conclusions

Data summarized in this review highlight the widespread occurrence at sea of *Penicillium/Talaromyces* strains, and their extraordinary potential as a source of novel bioactive compounds and drugs. As new data accumulate more and more, the awareness is increasing within the scientific community that these microorganisms represent a trove of unexplored biodiversity, and that more exhaustive investigations should be carried out. In this regard, a comprehensive work was recently published concerning diversity and antifungal properties of a group of 184 marine strains belonging to 36 different *Penicillium* species from Korea [216]. Also a comparison shows that as many as 18 of these species were not even mentioned in our review, which makes it very likely that our proposed record series will be considerably expanded if this biological material is further characterized in order to detect the biochemical determinants of the fungitoxic effects.

In the meantime, much work is to be done with reference to more complete characterization of the biological activities of the material accumulated so far, in view of the increasing number of products which can evolve to the drug level. A good example is represented by sorbicillactone A, whose notable antileukemic effects have stimulated studies for improving its laboratory yields in view of large scale production [217]. However, progress towards this ultimate step is largely dependent on the extent to which the pharmaceutical industry will prove to be prepared to grasp such a great opportunity. It is desirable that the recent policy adopted by most governments worldwide aimed at involving the manufacturing industry in funding for basic research, turns into a more decisive effort, with ensuing results, in this direction.

## Figures and Tables

**Table 1:** Secondary metabolites of *Penicillium* and *Talaromyces* strains of marine origin. List is based on the chronological order of isolation of the producing strains.

Species Name ^1^	Strain No.	Source of Isolation ^2^	Location	Products ^3^	References
*P. fellutanum* (*P. dierckxii*)	-	**F** *Apogon endekataenia*	Manazaru (Japan)	Fellutamide A–B	[28]
*Penicillium* sp. (*P. marinum*)	OUPS-79	**G** *Ulva (Enteromorpha) intestinalis*	Tanabe Bay (Japan)	Communesin A–B, Penochalasin A–H, Penostatin A–I, Chaetoglobosin A,F,O, Patulin, Epiepoxydon	[29,30,31,32,33,34]
*P. citrinum*	-	**S** unidentified sponge	Suruga Bay (Japan)	Cathestatin A–B, Estatin A–B	[35]
*Penicillium* sp.	BM1689-P	sediment	Uchiura Bay (Japan)	Epolactaene	[36]
*Penicillium* sp.	BM923	sediment	Miho (Japan)	Acetophthalidin, 3,4,6-Trihydroxymellein	[37]
*Penicillium* sp.	-	intertidal sediment	San Antonio Oeste (Argentina)	Cyclo(l-prolyl-l-tyrosyl)	[38]
*Penicillium* sp.	NI15501	sediment (depth 14 m)	Tomari (Japan)	NI15501A	[39]
*P. waksmanii*	OUPS-N133	**B** *Sargassum ringgoldianum*	Japan	Pyrenocine A–B,D–E, *Cis*-bis(methylthio)silvatin	[40]
*P. citrinum*	many strains	several sources	Mochima Bay and Paria Bay (Venezuela)	Citrinin, Tanzawaic acid A	[41,42]
*P. steckii*	M23B-7 = IBT20952 and 12 more strains	**T** unidentified tunicate, and other sources (molluscs, fish, sponges)	Mochima Bay and Paria Bay (Venezuela)	Tanzawaic acid E–F, 3,7-Dimethyl-8-hydroxy-6-methoxyisochroman, 3,7-Dimethyl-1,8-dihydroxy-6-methoxyisochroman	[41,42]
*Penicillium* sp.	#CNC-350	**G** *Avrainvillea longicaulis*	Sweetings Cay (Bahamas)	Verticillin A, 11′-Deoxyverticillin A, 11,11′-Dideoxyverticillin A, Bisdethio-bis(methylthio)-dioxopiperazine	[43]
*Penicillium* sp.	K029	**M** *Mytilus coruscus*	Seragaki (Japan)	Coruscol A, Herquline A	[44]
*Penicillium* sp.	K036	**M** *M. coruscus*	Seragaki (Japan)	Sculezonone A–B	[45]
*Penicillium* sp.	#386	sand	South China Sea	Penicillazine (Trichodermamide A)	[46]
*P.* cf. *montanense*	HBI-3/D	**S** *Xestospongia exigua*	Mangangan Island (Indonesia)	Xestodecalactone A–C	[47]
*P. citrinum*	991084	**S** *Axinella* sp.	Papua New Guinea	Isocyclocitrinol A, 22-Acetylisocyclocitrinol A	[48]
*P. brocae*	F97S76	**S** *Zyzzya* sp.	Fiji	Brocaenol A–C	[49]
*Penicillium* sp. (*P. dravuni*)	F01V25	**G** *Dictyosphaeria versluyii*	Dravuni (Fiji)	Dictyosphaeric acid A–B, Carviolin	[50]
*Penicillium* sp. (*P. marinum*)	E-00-12/3	**S** *Axinella verrucosa*	Elba Island (Italy)	Communesin B,C–D, Griseofulvin, Dechlorogriseofulvin, Oxaline	[51]
*P.* cf. *brevicompactum*	E-00-2/6a	**S** *Petrosia ficiformis*	Elba Island (Italy)	Petrosifungin A–B, Brevianamide A, Asperphenamate, Mycophenolic acid	[52]
*Penicillium* sp.	a004181, b004181	sediment (depth 4380 ft)	Matuka (Fiji)	Anserinone A–B, Formylanserinone B, Epoxyserinone A–B, Deoxyanserinone B, Hydroxymethylanserinone B	[53]
*P. waksmanii* (*Penicillium* sp.)	LCP99.43.43 = MMS351	water	La Prée (France)	Griseofulvin, Dechlorogriseofulvin, Orcinol, Penicillic acid, Agroclavine, Festuclavine, Nortryptoquivaline, Ligerin	[54,55,56]
*P. citrinum*	N059	**R** *Actinotrichia fragilis*	Okinawa (Japan)	Citrinin, Citrinadin A–B	[57,58]
*P. citrinum*	N055	**F** *Scarus ovifrons*	Okinawa (Japan)	Perinadine A, Scalusamide A–C	[59,60]
*P. janczewskii*	H-TW5/869	water	Helgoland Island (Germany)	3,4-Dihydroxy-4-(4′-methoxyphenyl)-3,4-dihydro-2(1H)-quinolinone, Peniprequinolone, 3-Methoxy-4-hydroxy-4-(4′-methoxyphenyl)-3,4-dihydro-2(1H)-quinolinone	[61]
*P. chrysogenum*	DSM16137 = E01-10/3	**S** *Ircinia fasciculata*	Elba Island (Italy)	Sorbicillactone A–B, Sorbivinetone, Sorbivinetol, Sorbifuranone A–C, Bisvertinolone, Sorbicillin, Oxosorbicillinol, Meleagrin, Roquefortine C–D	[62,63]
*P. terrestre* (*P. crustosum*)	M204077	sediment	Jiaozhou Bay (China)	Sorbicillin, Dihydrobisvertinolone, Tetrahydrobisvertinolone, Trichodimerol, Dihydrotrichodimerol, Tetrahydrotrichodimerol, Sorbiterrin A, Penicillone A–B, Chloctanspirone A–B, Terrestrol A–H,K–L, 2-(2′-3′-Dihydrosorbyl)-3,6-dimethyl-5-hydroxy-1,4-benzoquinone, 3-Acetonyl-2,6-dimethyl-5-hydroxy-1,4-benzoquinone	[64,65,66,67,68,69]
*P. janthinellum*	-	**C** *Dendronephyta* sp.	Hainan (China)	Griseofulvin, Dechlorogriseofulvin, Janthinolide A–B, Deoxymycelianamide	[70]
*P. brevicompactum*	Cl-2002	**S** *Cliona* sp.	Quintay (Chile)	Mycophenolic acid, Mycophenolic acid methyl ester, Tyrosol	[71]
*P. rugulosum* (*T. rugulosus*)	KF021	**S** *Chondrosia reniformis*	Elba Island (Italy)	Prugosene A1,A2,B1,B2,B3,C1,C2	[72]
*Penicillium* sp.	-	**B** *Sargassum tortile*	Toyama Bay (Japan)	4-Hydroxy-2-methoxyacetanilide, 4-Methoxyphenylacetic acid, 4-(2-Hydroxyethyl)phenol, 3-Methoxyphenol, 4-Hydroxyphenylacetic acid	[73]
*P. janthinellum*	-	sediment (depth 11 m)	Amursky Bay (Sea of Japan)	Shearinine A,D–F	[74]
*P. bilaiae*	MST-MF667	boat ramp	Port Huon (Tasmania, Australia)	Cyclo(l-prolyl-l-tyrosyl), Cyclo(l-phenalanyl-l-prolyl), Cyclo(l-prolyl-l-valyl), *Cis*-bis(methylthio)silvatin, Bilain A–C, Pistillarin, Citromycin, 2,3-Dihydrocitromycin, Citromycetin, 2,3-Dihydrocitromycetin	[75]
*Penicillium* sp.	MFA446	**G** *Ulva pertusa*	Bijin Island (Korea)	Citrinin, Citrinin H2, Redoxcitrinin, Phenol A, Phenol A acid, 4-Hydroxymellein	[76]
*P. aurantiogriseum*	SP0-19	**S** *Mycale plumose*	Jiaozhou Bay (China)	Aurantiomide A–C, Anacin	[77]
*P. stoloniferum* (*P. brevicompactum*)	QY2-10	**T** unidentified ascidian	Jiaozhou Bay (China)	Stoloniferol A–B	[78]
*P. flavidorsum* (*P. glabrum*)	SHK1-27	sediment	Weizhou Island (China)	Averufin, 8-*O*-Methylaverufin, 6,8-*O*-Dimethylaverufin, Averantin, Nidurufin, Versicolorin A–B, Versiconol	[79]
*P. minioluteum* (*T. minioluteus*)	03HE3-1	mud	Heita Bay (Japan)	Miniolutelide A–B, 22-Epoxyberkeleydione	[80]
*Penicillium* sp.	BL27-2	mud	Bering Sea	3-Acetyl-13-deoxyphomenone, 8α-Hydroxy-13-deoxyphomenone, Sporogen-AO1, 3-Acetyl-9,7(11)-dien-7α-hydroxy-8-oxoeremophilane, 6-Dehydropetasol, 7-Hydroxypetasol	[81]
*Penicillium* sp.	SS080624SCf1	**T** *Didemnum molle*	Ishigaki Island (Japan)	Phomenone, Sporogen-AO1, JBIR-27, JBIR-28	[82]
*Penicillium* sp. (*Talaromyces* sp.)	AF1-2	salt pan	Australia	3-*O*-Methylfunicone	[83]
*Penicillium* sp.	CANU MCPT14-1-5	**B** *Xiphophora gladiata*	Otago (New Zealand)	PF1140, Deoxy-PF1140, Deoxyakanthomycin	[84]
*Penicillium* sp.	i-1-1	**G** *Blidingia minima*	Yantai (China)	Citrinin, Citrinal A, 2,3,4-Trimethyl-5,7-dihydroxy-2,3-dihydrobenzofuran	[85]
*Penicillium* sp.	EG-51	**G** *Ulva* sp.	Suez Canal (Egypt)	Chromanone A	[86]
*Penicillium* sp.	F1	sediment (depth 5080 m)	Pacific Ocean	Brevicompanine B,D–H, Fructigenine B	[87]
*Penicillium* sp.	F23-2	sediment (depth 5080 m)	Pacific Ocean	Meleagrin B–E, Roquefortines F–I, Conidiogenone B–G, Sorbicillamine A–E, Bisvertinolone, Rezishanone C, Penicyclone A–E	[88,89,90,91]
*Penicillium* sp.	3A00005	sediment (depth 5115 m)	East Pacific Ocean	Brevione A–B,F–K, Sterolic acid	[92,93]
*P. expansum*	MMS42	sediment	Le Croisic (France)	Communesin A–B, D–F, Com470, Com512, Com522, Com524, Com570, Com622, Com 644, Patulin, Chaetoglobosin 528, Chaetoglobosin 530, Citrinin, Roquefortine C–D, Expansolide A–B, Aurantioclavine, Verruculotoxin	[54,94]
*Penicillium* sp.	PSU-F44	**C** *Annella* sp.	Similan Islands (Thailand)	Penicipyrone, Penicilactone, Brefeldin A,C, Oxobrefeldin A	[95]
*Penicillium* sp.	PSU-F40	**C** *Annella* sp.	Similan Islands (Thailand)	Penicipyrone, Penicipyranone, Penicisochroman A–E, Penicisoquinoline, Peniciphenol, TMC-120B, TMC-120C, 2-(2-Methoxybenzoyl)pyrrole, 1-(2,4-Dihydroxy-6-methylphenyl)-3-methyl-1-butanone, Nicotinic acid	[96]
*Penicillium* sp.	M207142	sediment	China	(2*E*,4*E*)-1-(2,6-Dihydroxy-3,5-dimethyl-phenyl)hexa-2,4-dien-1-one, Penicillone A, 2′,3′-Dihydrosorbicillin	[97]
*P. chrysogenum*	R03-8/4 = LF066	**S** *Tethya aurantium*	Limsky Canal (Croatia)	Meleagrin, Roquefortine C–D, Sorbifuranone B–C, Bisvertinolone, 2′,3′-Dihydrosorbicillin, Xanthocillins, Cillifuranone	[63,98]
*P. citrinum*	SpI080624G1f01	**S** unidentified Demospongia	Ishigaki Island (Japan)	Redoxcitrinin, Sclerotinin A–B, Bisorbibutenolide, Bisvertinolone, Trichodimerol, JBIR-59, JBIR-124	[99,100]
*P. oxalicum*	F30 = CBMAI1185	**G** *Caulerpa* sp.	Sao Paulo State (Brazil)	Meleagrin, Oxaline	[101]
*P. citrinum*	F53 = CBMAI1186	**G** *Caulerpa* sp.	Sao Paulo State (Brazil)	Citrinin, Citrinalin A–B, (*E*)-1-(2,3-dihydro-1H-pyrrol-1-yl)-2-methyldec-8-ene-1,3-dione, 1-(2,3-dihydro-1H-pyrrol-1-yl)-2-methyldecane-1,3-dione	[101]
*P. griseofulvum*	-	sediment (depth 2481 m)	Pacific Ocean	Echinulin, Preechinulin, Didehydroechinulin, Isoechinulin B, Neoechinulins A–B, Tardioxopiperazine A, Variecolorin H,M–O	[102]
*P. aurantiogriseum*	MF361	mud	Bohai Sea (China)	Verrucosidin, Norverrrucosidin, Verrucosidinol, Verrucosidinol acetate, Terrestric acid, Aurantiomide C, Auranthine, Auranomide A–C	[103,104]
*P. chrysogenum*	MFB574-2	**R** *Hypnea* species complex	Yokgee Island (Korea)	4,6,4′,6′-Tetrabromo-3,3′-dihydroxy-5,5′-dimethyldiphenyl ether, 4,6,2′,4′,6′-Pentabromo-3,3′-dihydroxy-5,5′-dimethyldiphenyl ether, 3,3′-Dihydroxy-5,5′-dimethyldiphenyl ether, Violacerol I–II	[105]
*Penicillium* sp.	CNL-338	**R** *Laurencia* sp.	Bahamas	Penilumamide, Aspochalasin D–E	[106]
*P. chrysogenum*	QEN-24S	**R** *Laurencia* sp.	Weizhou Island (China)	Penicitide A–B, Penicimonoterpene, Penicisteroid A–B, Conidiogenol, 2-(2,4-Dihydroxy-6-methylbenzoyl)-glycerol, Anicequol, 1-(2,4-Dihydroxy-6-methylbenzoyl)-glycerol, Conidiogenone B–D,F,H–I	[107,108,109]
*P. glabrum*	-	**P** *Zostera marina* (stem)	Trinity Bay (Sea of Japan)	Sulochrin, 4-Methoxy-3-methylgoniothalamin	[110]
*P. implicatum*	-	**P** *Z. marina* (rhizome)	Trinity Bay (Sea of Japan)	Sulochrin, 4-Methoxy-3-methylgoniothalamin	[110]
*P. citrinum*	-	sediment	Langqi Island (China)	Citrinin, Decarboxydihydrocitrinone, Penicitrinol C–E, Dicitrinone B, Penicitrinine A	[111,112,113]
*Penicillium* sp.	JMF034	sediment (depth 1151 m)	Suruga Bay (Japan)	Gliotoxin, Gliotoxin G, 5a,6-Didehydrogliotoxin, 6-Deoxy-5a,6-didehydrogliotoxin, Bis(dethio)-10a-methylthio-3a-deoxy-3,3a-didehydrogliotoxin, Bis(dethio)bis(methylthio)gliotoxin, Bis(dethio)bis-(methylthio)-5a,6-didehydrogliotoxin	[114]
*P. brevicompactum*	LF259	**S** *T. aurantium*	Limsky Canal (Croatia)	Mycophenolic acid	[98]
*P. citreoviride*	LF590	**S** *T. aurantium*	Limsky Canal (Croatia)	Citreoviridins, Territrem B	[98]
*P. canescens* (*Penicillium* sp.)	LF596	**S** *T. aurantium*	Limsky Canal (Croatia)	Griseofulvin, Fiscalin A–C, Tryptoquivalin, Nortryptoquivalin	[98]
*P. sclerotiorum*	LF607	**S** *T. aurantium*	Limsky Canal (Croatia)	Sclerotiorin, Sclerotioramin, Azaphilone derivative (comp. D)	[98]
*Penicillium* sp.	J05B-3-F-1	**S** *Stelletta* sp.	Jeju Island (Korea)	(3*S*)-Hexylitaconic acid, (3*S*,8*R*)-Methyl 8-hydroxy-3-methoxycarbonyl-2-methylenenonanoate, (3*S*,8*R*)-8-Hydroxy-3-carboxy-2-methylenenonanoic acid, (3*S*)-9-Hydroxy-3-carboxy-2-methylenenonanoic acid, (3*S*)-Methyl-9-hydroxy-3-methoxycarbonyl-2-methylenenonanoate	[115]
*P. paneum*	SD-44	sediment (depth 20 m)	South China Sea	Penipanoid A–C, 2-(4-Hydroxybenzyl)quinazolin-4(3H)-one, Penipacid A–E, Penipaline A–C, (−)-(3*S*)-2,3,4,9-Tetrahydro-1,1-dimethyl-1H-β-carboline-3-carboxylic acid, 1,7-Dihydro-7,7-dimethylpyrano[2,3-*g*]indole-3-carbaldehyde	[116,117,118]
*P. commune*	QSD-17	sediment	South China Sea	Meleagrin, Asperamide B1, Citreohybridonol, 3-Deacetylcitreohybridonol, Comazaphilone A–F, Isophomenone, Conidiogenone B–D,F, Conidiogenol	[12,119]
*Penicillium* sp.	DG(M3)6′C	**C** *Didemnum granulatum*	Toque Island (Brazil)	13-Desoxyphomenone	[120]
*P. raistrickii*	AC(M2)14	**S** *Axinella* cf. *corrugata*	Toque Island (Brazil)	Norlichexanthone	[120]
*P. paxilli*	Ma(G)K	**S** *Mycale angulosa*	Toque Island (Brazil)	Pyrenocine A–B,J	[120]
*P. steckii*	AS(F)39	**B** *Sargassum* sp.	Toque Island (Brazil)	8-Methoxy-3,5-dimethylisochroman-6-ol	[120]
*Penicillium* sp.	ghq208	sediment	Jiaozhou Bay (China)	Penicinoline, Penicinoline E, Methylpenicinoline, Quinolactacide	[121]
*P. pinophilum* (*T. pinophilus*)	-	**G** *Ulva fasciata*	Kasai Marine Park (Japan)	Pinophilin A–B, Sch725680	[122]
*Penicillium* sp.	fS36	**S** unidentified sponge	Takarajima Island (Japan)	JBR-113,-114,-115	[123]
*Penicillium* sp.	F00120	sediment (depth 1300 m)	South China Sea	Penicilliumin A	[9]
*Penicillium* sp.	SOF07	sediment (depth 675 m)	South China Sea	Mycophenolic acid, Hydroxy-mycophenolic acid, Penicacid A–C	[124]
*P. crustosum*	PRB-2	sediment (depth 526 m)	Prydz Bay (Antarctica)	Penilactone A–B, 2′,4′-Dihydroxy-3′-methoxymethyl-5′-methylacetophenone	[125]
*P. commune*	SD-118	sediment	South China Sea	Meleagrin, Chrysogine, Methyl 2-*N*-(2-hydroxyphenyl)carbamoylacetate, Asperamide A–B, Xanthocillin X, *N*-(2-Hydroxypropanoyl)-2-amino benzoic acid amide, *N*-(2-Hydroxyphenyl)acetamide, 4-Hydroxy benzaldehyde, Methyl-2-(2-(1H-indol-3-yl)ethyl)carbamoyl)acetate, *N*2′-Acetyltryptophan methyl ester, *N*-Acetyldopamine	[126]
*P. commune*	518	**C** *Muricella abnormalis*	Danzhou (Hainan, China)	Communol A–G, Clavatol, 2,4-Dihydroxy-3-methylacetophenone, 2,4-Dihydroxy-3-methoxymethyl-5-methylacetophenone, 2,4-Dihydroxy-5-methylacetophenone, *cis*-Bis(methylthio)silvatin	[127]
*P. canescens*	MMS194	water	La Baule (France)	Griseofulvin, Dechlorogriseofulvin, Oxaline, Maculosin, Penicillic acid, Penitremone A–C	[54]
*P. canescens*	MMS460	sediment	Le Croisic (France)	Griseofulvin, Dechlorogriseofulvin, Oxaline, Penicillic acid, Penitremone A–C	[54]
*Penicillium* sp.	MMS747	sediment	La Couplasse (France)	Griseofulvin, Dechlorogriseofulvin, Penicillic acid, Nortryptoquivaline, Agroclavine, Festuclavine	[54]
*P. chrysogenum*	MMS5	**M** shellfish	Le Croisic (France)	Meleagrin, Roquefortine C–D, Chrysogine, Aurantioclavine, Maculosin, Glandicolin A–B, Terrestric acid, Verruculotoxin	[54]
*P. antarcticum*	MMS14	**M** cockles	Le Croisic (France)	Chrysogine, Cladosporin(=asperentin), 5-Hydroxyasperentin, Antarone A, Violaceic acid, Patulin, Terrestric acid	[54,128]
*P. antarcticum*	MMS15	**M** cockles	Le Croisic (France)	Chrysogine, Cladosporin, 5-Hydroxyasperentin, Aurantioclavine, Antarone A, Patulin, Terrestric acid	[54,128]
*P. antarcticum*	MMS163	**M** mussel	Loire estuary (France)	Patulin, Chrysogine, Cladosporin, 5-Hydroxyasperentin, Terrestric acid	[128]
*P. marinum*	MMS266	**M** mussel	La Baule (France)	Penostatin derivatives, Fusoxysporone	[128]
*P. restrictum*	MMS417	**M** cockles	Le Croisic (France)	Pestalotin, Hydroxypestalotin, 5,6-Dihydro-4-methoxy-6-(1-oxopentyl)-2H-pyran-2-one	[128]
*P. citrinum*	-	**C** soft coral	Van Phong Bay (Vietnam)	JBIR-27, Petasol, Sporogen AO-1, Dihydrosporogen AO-1	[129]
*Penicillium* sp.	F011	sediment	Korea	Herqueiazole, Herqueioxazole, Herqueidiketal	[130]
*Penicillium* sp.	FF001	**S** *Melophlus* sp.	Cicia (Fiji)	Citrinin	[131]
*P. pinophilum* (*T. pinophilus*)	SD-272	sediment	Pearl River estuary (China)	Pinodiketopiperazine A, 6,7-Dihydroxy-3-methoxy-3-methyl phthalide, Cyclo(d-prolyl-d-valyl), Cyclo(*trans*-4-OH-d-prolyl-d-phenylalanyl), *N*-methylphenyldehydroalanyl-l-prolin-anhydrid, l-5-Oxoproline methyl ester, Rubralide C, Alternariol 2,4-dimethyl ether, Altenuene, 5′-Epialtenuene	[132]
*Penicillium* sp.	-	**B** *Fucus spiralis*	Shetland Islands (Scotland)	Patulin, Epiepoformin, Phyllostine, Cladosporin	[133]
*Penicillium* sp.	MWZ14-4	**S** unidentified sponge	Weizhou (South China Sea)	Penicimarin A–F, Penicifuran A–D, Aspergillumarin A–B, Sescandelin-B, 5,6,8-Trihydroxy-4-(1′-hydroxyethyl)isocoumarin	[134]
*Penicillium* sp.	SCSIO00258	**C** *Dichotella gemmacea*	Sanya (Hainan, China)	Penilloid A, Roquefortine C, Isoroquefortine C, Methoxyroquefortine C, Meleagrin, Glandicoline B, Neoxaline, (*Z*)-3-(1H-Imidazole-4-yimethylene)-6-(1H-indl-3-ylmethyl)-2,5–piperazinediol	[135]
*Penicillium* sp.	SCSGAF0023	**C** *D. gemmacea*	Sanya (Hainan, China)	Paecilin C, 6,8,5′6′-Tetrahydroxy-3′-methylflavone, Emodin, Citrorosein, Isorhodoptilometrin, Penicillixanthone A, Secalonic acid B–D	[136]
*Penicillium* sp.	SF-5203	intertidal sediment	Wan Island (Korea)	Fructigenine A, Cyclopenol	[137]
*Penicillium* sp.	SF-5292	**Z** unidentified Bryozoan	Jeju Island (Korea)	Penicillinolide A, Cycloexpansamine A–B	[138,139]
*Penicillium* sp.	SF-5295	**S** unidentified sponge	Jeju Island (Korea)	Viridicatol	[137]
*Penicillium* sp. (*P. glabrum*)	JF-55	**S** unidentified sponge	Jeju Island (Korea)	Penstyrylpyrone, Anhydrofulvic acid, Citromycetin	[140]
*Penicillium* sp.	JF-72	**S** unidentified sponge	Jeju Island (Korea)	Deoxyisoaustamide, Deoxydihydroisoaustamide, 16β-Hydroxy-17β-methoxy-deoxydihydroisoaustamide	[141]
*P. chrysogenum*	EN-118	**B** *Sargassum pallidum*	Fujian (China)	Chrysotriazole A–B, 2-(4-Hydroxybenzoyl)-4(3H)-quinazolinone, 2-(4-Hydroxybenzyl)quinazolin-4(3H)-one), 2-(4-Hydroxyphenyl)acetylamide), *N*-(2-(4-Hydroxyphenyl)acetyl)formamide, *N*-(2*E*)-(4-Hydroxyphenyl) ethenyl)formamide, *N*-(2*Z*)-(4-Hydroxyphenyl)ethenylformamide	[142]
*Penicillium* sp.	ZLN29	sediment	Jiaozhou Bay (China)	Penicillide, Prenpenicillide, Prenxanthone, Bioxanthracene, NG-011, NG-012, 15-G256α-2, 15-G256β	[143]
*Penicillium* sp.	F37	**S** *A. corrugata*	Arvoredo Island (Brazil)	*cis*-Cyclo(leucyl-tyrosyl)	[144]
*Penicillium* sp.	PR19N-1 = MBC06294	sludge (depth 1000 m)	Prydz Bay (Antarctica)	1-Chloro-3β-acetoxy-7-hydroxy-trinoreremophil-1,6,9-trien-8-one, 1-α-Chloro-2β-hydroxyeremophil-7(11),9-dien-8-one, 1α-Chloro-2β-hydroxyeremophil-7(11),9-dien-8-one, 5 new eremophilane compounds, Eremofortine C	[145,146]
*P. citrinum*	SCSGAF167	**C** *Echinogorgia aurantiaca*	Sanya (Hainan, China)	Penicitrinol G–H, 2,11-Dihydroxy-1-methoxycarbonyl-9-carboxylxanthone, Chrysophanol	[147]
*P. brefeldianum*	SD-273	sediment (depth 100 m)	Pearl River estuary (China)	Verruculogen, 24-Hydroxyverruculogen, 26-Hydroxyverruculogen, 13-*O*-Prenyl-26-hydroxyverruculogen, Fumitremorgin A, Cyclotryprostatin A, TR-2	[148]
*P. commune*	366606	water	Qingdao (China)	Penicilliumine	[149]
*P. echinulatum*	pt-4	**R** *Chondrus ocellatus*	Pingtan Island (China)	Arisugacin C,G,J,K, Territrem C	[150]
*Penicillium* sp.	F446	sediment (depth 25 m)	Geomun-do Island (Korea)	Penicillipyrone A–B	[151]
*T. trachyspermus*	KUFA0021	**S** *Clathria reianwardii*	Kram Island (Thailand)	Spiculisporic acid E, Glaucanic acid, Glauconic acid	[152]
*P. chrysogenum*	PJX-17	sediment	South China Sea	Sorbicathecol A–B, Protocatechuic acid methyl ester, Caffeic acid methyl ester	[153]
*Penicillium* sp.	SF-5995	**C** unidentified soft coral	Terra Nova Bay (Antarctica)	Methylpenicinoline	[154]
*P. adametzioides*	AS-53	**S** unidentified sponge	Wenchang (Hainan, China)	Lapatin A–B, Prelapatin B, N-Formyllapatin A, Glyantrypine, Adametizine A–B, Adametacorenol A–B, Peniciadametizine A–B, Brasiliamide A, Viridicatumtoxin, Fumitremorgin B, Verruculogen	[155,156,157]
*Penicillium* sp.	SF-6013	**U** *Brisaster latifrons*	Sea of Okhotsk (Russia)	Tanzawaic acid A–B,D–E, 2E,4Z-Tanzawaic acid D	[158]
*P. bialowiezense*	IBT28294	water	North Sea	Asperphenamates, Mycophenolic acid, F13459, Andrastin A, Chrysogeside B–E, Quinolactacin A, Raistrick phenols, Xanthoepocin, Citreohybridonol, Preaustinoids, Fellutamides, Breviones	[159]
*P. lividum*	KMM4663	**B** *Sargassum miyabei*	Lazurnaya Bay (Sea of Japan)	Sargassopenilline B–G	[160]
*P. thomii*	KMM4645	**B** *S. miyabei*	Lazurnaya Bay (Sea of Japan)	Sargassopenilline A,E	[160]
*Talaromyces* sp.	LF458	**S** *A. verrucosa*	Elba Island (Italy)	Talaromycesone A–B, Talaroxanthenone, Vermixocin A–B, AS-186c, Δ1′,3′,-1′-Dehydroxypenicillide, 1′,2′-Dehydropenicillide, 3′-Methoxy-1′2′-dehydropenicillide	[161]
*Talaromyces* sp.	SBE-14	**C** *Subergorgia suberosa*	Weizhou (South China Sea)	Talaromycin A-C^4^, Penicillide, Δ1′,3′,-1′-Dehydroxypenicillide,Purpactin A,C,C′, Tenellic acid methyl esther	[162]
*P. pinophilum* (*T. pinophilus*)	XS-20090E18	**C** unidentified gorgonian	Xisha Island (South China Sea)	Purpactin A, Penicillide, Isopenicillide, Hydroxypenicillide, Sch1385568, Sch725680, Pinophilin B,D–F, Mitorubrin, Mitorubrinol, Mitorubrinic acid	[163]
*T. miniolouteus*	PILE14-5	**S** unidentified sponge	Phi Phi Island (Thailand)	Minioluteumide A–D, Purpuride, Purpuride B, Berkedrimane B	[164]
*P. claviforme* (*P. vulpinum*)	KMM4665	**P** *Z. marina*	Peter the Great Gulf (Russia)	3-[2′(R)-Hydroxybutyl]-7-hydroxyphthalide, (–)-3-Butyl-7-hydroxyphthalide, Isopatulin, Cyclopenin, Cyclopeptin	[165]
*P. vinaceum*	CYE-88	**S** *Hyrtios erectus*	Yanbu (Saudi Arabia)	Penicillivinacine, Cyclo(d-tryptophanyl-l-prolyl), Citreoisocoumarin, Brevianamide F, Indol-3-carbaldehyde, α-Cyclopiazonic acid, Terretrione A	[166]
*Penicillium* sp.	CYE-87	**T** *Didemnum* sp.	Suez Canal (Egypt)	Terretrione C–D, Indol-3-carbaldehyde, 3,6-Diisobutylpyrazin-2(1H)-one, Methyl-2-([2-(1H-indol-3-yl)ethyl]carbamoyl)acetate, Tryptamine	[167]
*Penicillium* sp.	IO1	**S** *Ircinia oros*	Kermer (Turkey)	Fusarielin I, Griseofulvin, Dechlorogriseofulvin	[16]
*Penicillium* sp.	IO2	**S** *I. oros*	Kermer (Turkey)	Curvularin, Dehydrocurvularin, Trichodimerol	[16]
*P. expansum*	Y32	water	Indian Ocean, west of Sumatra	Communesine A–B,I, Fumiquinazoline Q, Prelapatin B, Penochalasin E, Glyantripine, Protuboxepin A–B,E, Cottoquinazoline A, Chaetoglobosin C	[168]
*Penicillium* sp.	KCB12F005	sediment	Haenam (Korea)	Haenamindole	[169]
*Penicillium* sp.	CF07370	sediment (depth 100 m)	Gulf of California (Mexico)	Tanzawaic acid B,E,M–P	[170]
*Penicillium* sp.	TPU1271	organic debris attached to oyster	Oshika Peninsula (Japan)	Penicyrone A–B, Verrucosidin, Fructigenine A, Verrucofortine, Cyclo(l-Tryptophanyl-l-Phenylalanyl), Cyclopenol, Cyclopenin, Penipratynolene, Aspterric acid, Viridicatol	[171]
*P. concentricum*	ZLQ-69	water	Bohai Sea (China)	Phenylpyropene B-D,E–F, Pyripyropene A–B,E,O	[172]
*P. verruculosum* (*T. verruculosum*)	TPU1311	**T** *Polycarpa aurata*	Manado (Indonesia)	Verruculide A–B, Chrodrimanins A–B,H	[173]

^1^ Current species name is specified in parentheses if different from the one reported in the original reference; ^2^ Information concerning the kind of organism is indicated as follows: **B** = brown alga; **C** = coral, soft coral; **F** = fish; **G** = green alga; **M** = mollusc; **P** = Angiosperm plant; **R** = red alga; **S** = sponge; **T** = tunicate; **U** = urchin; **Z** = bryozoan; ^3^ Products originally characterized from the corresponding strain are underlined; ^4^ Talaromycins A–C have been reported as new products. However, the same name was previously used for compounds with a different structure isolated from terrestrial *Talaromyces* strains [174,175].

**Table 2:** Novel compounds produced by marine *Penicillium/Talaromyces* strains displaying inhibitory properties against mammalian tumor cell lines.

Compound	Bioactivity	Cell Lines Assayed	References
Acetophthalidin	Cytostatic (arrest at M phase)	tsFT210	[37]
3-Acetyl-9,7(11)-dien-7α-hydroxyl-8-oxoeremophilane	Cytotoxic	A549, BEL-7402	[81]
Adametacorenol B	Cytotoxic	NCI-H446	[155]
Auranomides	Antiproliferative	K562, ACHN, HepG2, A549	[104]
Aurantiomide B	Cytotoxic	HL-60, P388	[77]
Aurantiomide C	Cytotoxic	BEL-7402, P388	
Breviones F–H	Antiproliferative	HeLa	[92]
Brevione I	Cytotoxic	MCF-7, A549	[93]
Brocaenols A–C	Cytotoxic	HCT-116	[49]
Chloctanspirones A–B	Cytotoxic	HL-60, A549	[69]
1-Chloro-3β-acetoxy-7-hydroxyl-trinoreremophil-1,6,9-trien-8-one	Cytotoxic	HL-60, A549	[145]
Citrinadin A	Cytotoxic	L1210, KB	[57]
Citrinadin B	Cytotoxic	L1210	[58]
Citrinal A	Cytotoxic	K562	[85]
Comazaphilones D–F	Cytotoxic	SW1990	[12]
Communesins A–B	Cytotoxic	P388	[29]
Communesins B–D	Antiproliferative	U-937, THP-1, NAMALWA, MOLT-3, SUP-B15	[51]
Conidiogenone B	Cytotoxic	BEL-7402, HL-60	[88]
11′-Deoxyverticillin A, 11,11′-Dideoxyverticillin A	Cytotoxic	HCT-116	[43]
Dihydrotrichodimerol, Tetrahydrotrichodimerol	Cytotoxic	P388, A549	[66]
(2*E*,4*E*)-1-(2,6-Dihydroxy-3,5-dimethylphenyl)hexa-2,4-dien-1-one	Cytotoxic	HeLa, SW620	[97]
3,4-Dihydroxy-4-(4′-methoxyphenyl)-3,4-dihydro-2(1H)-quinolinone	Cytotoxic	SKOV-3	[61]
Epolactaene	Cytostatic (arrest at G_0_/G_1_ phase)	SH-SY5Y	[204]
Fellutamides A–B	Cytotoxic	P388, KB	[28]
Formylanserinone B, Epoxyserinone B	Cytotoxic	L1210, C38, CFU-GM, H116, H125, MDA-MB-435	[53]
Gliotoxin derivatives	Cytotoxic	P388	[114]
Herqueidiketal	Cytotoxic	A549	[130]
JBIR-28	Cytotoxic	HeLa	[82]
Ligerin	Antiproliferative	OSRGa, POS1, AT6-1, L929	[56]
Meleagrin B	Cytotoxic	BEL-7402, HL-60, A549, MOLT-4	[88]
	Pro-apoptotic	HL-60	[89]
Meleagrins D–E	Cytotoxic	A549	[89]
Minioluteumides	Cytotoxic	HepG2	[164]
Penicacid A	Antiproliferative	Mouse splenocytes	[124]
Penicilliumin A	Cytotoxic	A375, B16, HeLa	[9]
Penicillivinacine	Antimigratory	MDA-MB-231	[166]
Penicillones A–B	Cytotoxic	P388, A549	[68]
Penicillone A	Cytotoxic	SW620	[97]
Penicisteroid A	Cytotoxic	HeLa, SW1990, NCI-H460	[108]
Penicitide A	Cytotoxic	HepG2	[107]
Penicitrinine A	Antiproliferative, pro-apoptotic	23 tumor cell lines	[111]
Penicitrinols C, E	Cytotoxic	HL-60	[112]
Penipacids A, E	Cytotoxic	RKO	[117]
Penipalines B–C	Cytotoxic	A549, HCT-116	[118]
Penipanoid A	Cytotoxic	SMMC-7721	[116]
Penochalasins A–H	Cytotoxic	P388	[30,34]
Penostatins A–C,E–I	Cytotoxic	P388	[31,32,33]
Perinadine A	Cytotoxic	L1210	[59]
Phenylpyropene E	Cytotoxic	MGC-803	[171]
Pinophilins	Antiproliferative	A549, BALL-1, HCT116, HeLa, NUGC-3	[122]
Prenpenicillide	Cytotoxic	HepG2	[143]
Pyrenocine E	Cytotoxic	P388	[40]
Sargassopenilline E	Cytotoxic	CD-1	[160]
Shearinines	Pro-apoptotic	HL-60	[74]
Sorbicillactones, Sorbivinetol, Sorbivinetone	Cytotoxic	L5178y	[62]
Tanzawaic acids	Antiproliferative, pro-apoptotic	K562, U937, Jurkat, Raji	[170]
Terrestrols A–H	Cytotoxic	BEL-7402, HL-60, A549, MOLT-4	[64]
Terretrione D	Antimigratory	MDA-MB-231	[167]

**Table 3:** Novel antibiotic compounds produced by marine *Penicillium/Talaromyces* strains.

Compound	Bioactivity	Microbial Species Assayed	References
Adametizine A	Antibacterial	*Aeromonas hydrophila*, *Staphyloccocus aureus*, *Vibrio harveyi*, *Vibrio parahaemolyticus*	[155]
	Antifungal	*Gaeumannomyces graminis*	
Arisugacin K	Antibacterial	*Escherichia coli*	[150]
Cillifuranone	Antibacterial	*Xanthomonas campestris*	[98]
	Antifungal	*Septoria tritici*	
Comazaphilones	Antibacterial	*Bacillus subtilis*, *Pseudomonas fluorescens*, *S. aureus* m.r.	[12]
Communol A, F–G	Antibacterial	*Enterobacter aerogenes*, *E. coli*	[127]
Conidiogenone B	Antibacterial	*Pseudomonas aeruginosa*, *Pseudomonas fluorescens*, *S. aureus* m.r., *Staphylococcus epidermidis*	[109]
	Antifungal	*Candida albicans*	
Dictyosphaeric acid A	Antibacterial	*Enterococcus faecium*, *S. aureus*, *S. aureus* m.r.	[50]
	Antifungal	*C. albicans*	
Isocyclocitrinols	Antibacterial	*Enterococcus durans*, *S. epidermidis*	[48]
Peniciadametizines	Antifungal	*Alternaria brassicae*	[156]
Penicifuran A	Antibacterial	*Bacillus cereus*, *Staphylococcus albus*	[134]
Penicilactone	Antibacterial	*S. aureus* m.r.	[95]
Penicimonoterpene	Antifungal	*A. brassicae*, *Aspergillus niger*, *Fusarium graminearum*	[107,214]
	Antibacterial	*A. hydrophila*, *E. coli*, *Micrococcus luteus*, *S. aureus*, *V. harveyi*, *V. parahaemolyticus*	[214]
Penicisteroid A	Antifungal	*A. brassicae*, *A. niger*	[108]
Penicitide A	Antifungal	*A. brassicae*, *A. niger*	[107]
Penicyclones A–E	Antibacterial	*S. aureus*	[91]
Perinadine A	Antibacterial	*B. subtilis*, *M. luteus*	[59]
Pinodiketopiperazine A	Antibacterial	*E. coli*	[132]
Scalusamide A	Antibacterial	*M. luteus*	[60]
	Antifungal	*Cryptococcus neoformans*	
Talaromycesones	Antibacterial	*S. aureus* m.r., *S. epidermidis*	[161]
Terretrione D	Antifungal	*C. albicans*	[167]
Xestodecalactone B	Antifungal	*C. albicans*	[47]

m.r.: Methicillin resistant.
